# An epidemiological study on the factors including genetic polymorphism influencing ALT >30 U/L and liver fibrosis progression in metabolic dysfunction‐associated steatotic liver disease among the general population

**DOI:** 10.1002/jgh3.70043

**Published:** 2024-12-19

**Authors:** Satoshi Sato, Chikara Iino, Takafumi Sasada, Go Soma, Keisuke Furusawa, Kenta Yoshida, Kaori Sawada, Tatsuya Mikami, Shinsaku Fukuda, Shigeyuki Nakaji, Hirotake Sakuraba

**Affiliations:** ^1^ Department of Gastroenterology, Hematology and Clinical Immunology Hirosaki University Graduate School of Medicine Hirosaki Japan; ^2^ Department of Preemptive Medicine Hirosaki University Graduate School of Medicine Hirosaki Japan

**Keywords:** cardiovascular metabolic risk factors, lifestyle, liver fibrosis, metabolic dysfunction‐associated steatotic liver disease, single‐nucleotide polymorphisms

## Abstract

**Background and Aim:**

Identifying the factors contributing to the progression of metabolic dysfunction‐associated steatotic liver disease (MASLD), a lifestyle‐related disease, is crucial for preventing future liver‐related deaths. This study aimed to epidemiologically investigate factors, including single‐nucleotide polymorphisms (SNPs) associated with alanine aminotransferase (ALT) levels >30 U/L and potential risk factors for liver fibrosis, in a general population cohort of patients with MASLD.

**Methods:**

Among 1059 participants in the health checkup project, 228 who were diagnosed with MASLD were analyzed. Liver fat content and stiffness were measured using FibroScan, and 13 SNPs associated with non‐alcoholic fatty liver disease (NAFLD) were measured in addition to other clinical parameters.

**Results:**

In the multivariate analysis, male sex, younger age, and high triglyceride levels were significant risk factors for ALT levels >30 U/L (*P*‐value < 0.05). Furthermore, among the 13 SNPs measured, only the GG genotypes of patatin‐like phospholipase domain‐containing 3 gene (PNPLA3) rs738409 and rs2896019 were significant risk factors for ALT levels >30 U/L (*P*‐value 0.004 and 0.007). The GG genotypes of PNPLA3 rs738409 and rs2896019 had higher FibroScan‐aspartate aminotransferase (FAST) and APRI scores than the CC + CG and TT + TG genotypes (*P*‐value < 0.05). In addition, multivariate analysis revealed that the GG genotypes of rs738409 and rs2896019 were significant risk factors independent of cardiovascular metabolic risk for patients with MASLD (*P*‐value 0.038 and 0.021).

**Conclusion:**

An individualized treatment approach is warranted for patients with MASLD due to the influence of various factors on its progression, including genetic factors and lifestyle diseases.

## Graphical abstract

In a general population cohort of patients with metabolic dysfunction‐associated steatotic liver disease (MASLD) patients, male sex, younger age, high triglyceride levels, and GG genotypes of patatin‐like phospholipase domain‐containing 3 gene (PNPLA3) rs738409 and rs2896019 were significant factors influencing alanine aminotransferase levels > 30 U/L. Furthermore, GG genotypes of PNPLA3 rs738409 and rs2896019 were factors for liver fibrosis (FibroScan‐aspartate aminotransferase score > 0.35) independent of cardiometabolic criteria. An individualized treatment approach is warranted for patients with MASLD.

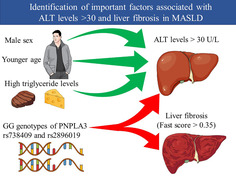

## Introduction

Metabolic dysfunction‐associated steatotic liver disease (MASLD) is recognized as a hepatic manifestation of metabolic syndrome, with a global prevalence of approximately 30%.^1^ In 2023, the term non‐alcoholic fatty liver disease (NAFLD) was changed to MASLD, and the diagnostic criteria were expanded to include the presence of at least one cardiovascular metabolic risk factor in addition to fatty liver.^2^

Alanine aminotransferase (ALT) is a liver‐specific enzyme that serves as a marker for several chronic liver diseases, including MASLD. Elevated ALT levels are a strong risk factor for NAFLD.^3^, ^4^ To address these concerns, the Nara Declaration was proposed in June 2023 at the 59th Annual Meeting of the Japanese Society of Hepatology, which calls for both healthcare professionals and the general public to be informed that medical attention should be sought if ALT levels exceed 30 U/L. However, several patients with NAFLD/non‐alcoholic steatohepatitis (NASH) do not exhibit elevated ALT levels.^5^ A meta‐analysis reported that 25% and 19% patients with NAFLD and NASH, respectively, had ALT levels within the normal range.^6^ Elevated ALT levels reflect severe liver inflammation and are associated with advanced liver fibrosis.^7^, ^8^ Additionally, ALT levels correlate with liver stiffness measurement (LSM) levels, suggesting that ALT is a useful marker for assessing the progression of liver fibrosis.^8^

Genetic factors are known to contribute to MASLD, which is a lifestyle‐related disease. In recent years, genome‐wide association studies (GWAS) have identified several single‐nucleotide polymorphisms (SNPs) in NAFLD susceptibility genes. In 2008, Romeo et al. identified one in the patatin‐like phospholipase domain‐containing 3 gene (PNPLA3).^9^ The SNP rs738409 (C > G) in PNPLA3 is associated with NAFLD in several ethnic groups, including Japanese individuals.^10^, ^11^, ^12^, ^13^, ^14^ Additionally, rs2896019 (T > G) in PNPLA3 is associated with fatty liver.^10^, ^15^, ^16^, ^17^ Moreover, various other SNPs are reportedly associated with NAFLD.^18^, ^19^, ^20^, ^21^, ^22^, ^23^, ^24^, ^25^, ^26^, ^27^, ^28^, ^29^

Previous studies have used invasive liver biopsies to evaluate liver fibrosis; however, FibroScan has recently enabled the noninvasive evaluation of liver fibrosis. The FibroScan‐aspartate aminotransferase (FAST) score is a simple algorithm developed to diagnose NASH with advanced fibrosis without liver biopsy.^30^ Despite the introduction of several noninvasive liver fibrosis assessment tools using FibroScan, the FAST score is most reliable.^31^ Moreover, although the FAST score was developed to diagnose NASH, its utility extends to the evaluation of metabolic dysfunction‐associated fatty liver disease (MAFLD) in the general population.^32^

Identifying risk factors for ALT levels >30 U/L in the MASLD population is important but not well elucidated. It is also important to investigate the factors that can lead to MASLD with ALT levels ≤30 U/L in order to prevent missing MASLD patients during health checkups and other medical examinations. As mentioned above, the development and progression of MASLD involve not only sex, age, and lifestyle but also genetic factors; however, only a few studies have examined them together. Furthermore, in addition to elevated ALT levels, which are a risk factor for liver fibrosis, cardiovascular metabolic risk factors, which are diagnostic criteria for MASLD, are also known to cause liver fibrosis. However, few epidemiological studies have examined the association between genetic polymorphisms and liver fibrosis while considering the influence of cardiovascular metabolic risk factors.^33^, ^34^, ^35^

Therefore, this study aimed to epidemiologically investigate the factors associated with ALT levels >30 U/L and the potential risk factors for liver fibrosis, including genetic polymorphisms, in a general population cohort of patients with MASLD.

## Methods

### Study participants

**Figure 1:**
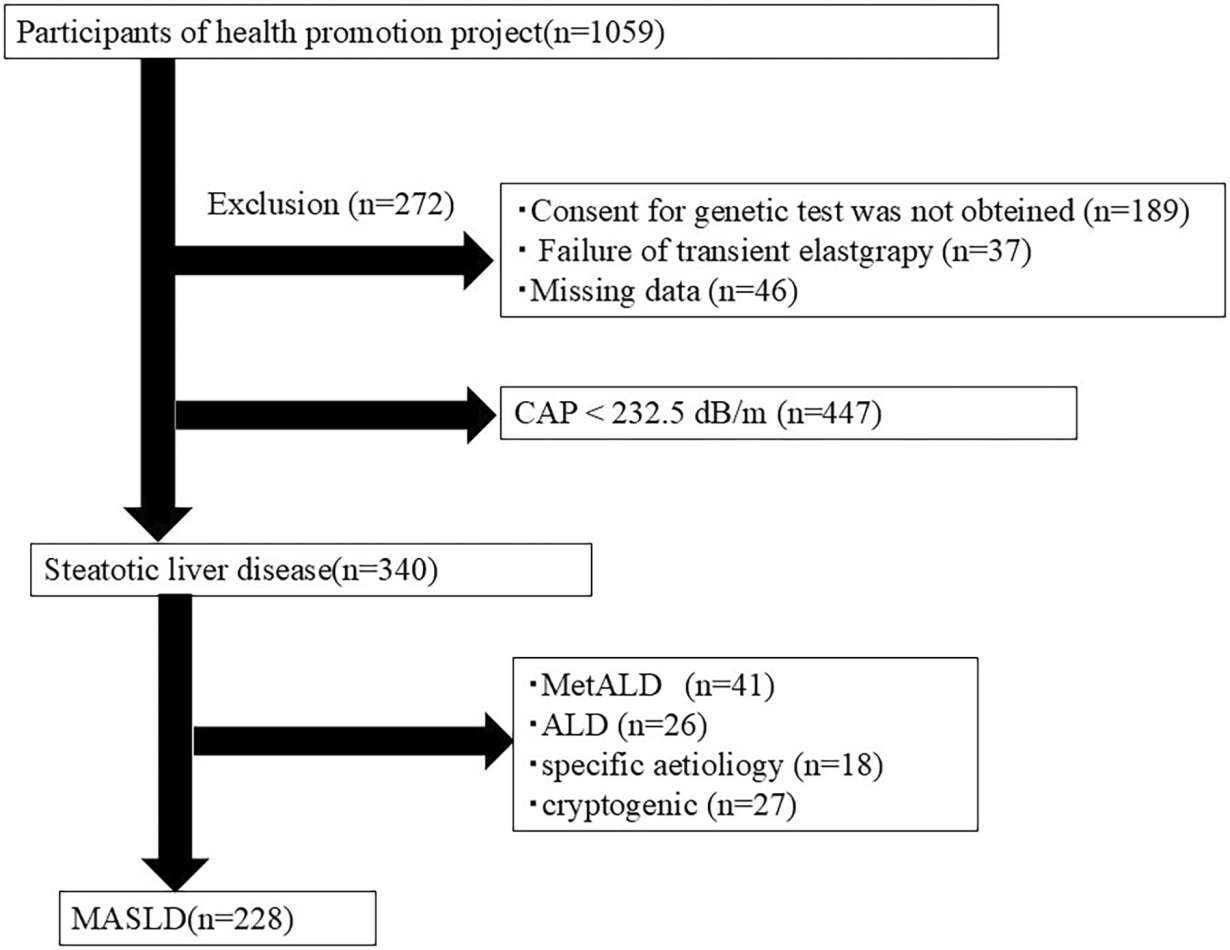
Study enrollment flowchart.

This study was conducted as part of the Iwaki Health Promotion Project, which is a community‐based health promotion project for the general Japanese population. It is conducted annually in June as a regular health checkup for residents of the Iwaki area of Hirosaki City, Aomori Prefecture.^36^ All the participants voluntarily responded to a public call for participation. A total of 1059 adults (aged 19–88 years) participated in this project. After excluding participants who did not consent to genetic testing, those whose transient elastography could not be accurately measured, and those with missing values, 340 individuals with steatotic liver disease, with a CAP value of 232.5 dB/m or higher on the FibroScan, were included in the study. The breakdown of steatotic liver disease was as follows: 228 patients with MASLD, 41 patients with metabolic dysfunction‐associated alcoholic liver disease, 26 patients with alcoholic liver disease, 18 patients with specific etiology, and 27 patients with cryptogenic steatotic liver disease. We further analyzed the 228 patients with MASLD (Fig. 1).

### Transient elastography

CAP and LSM were performed using a Fibroscan530 (Echosens, Paris, France) using the M and XL probes. All examinations were performed by five hepatologists who underwent specialized training. When the number of measurements was <10 or the ratio of the interquartile range was >0.30, the measured values were excluded because of unreliability. According to previous studies, steatosis was defined as a CAP value >232.5 dB/m.^37^

### Clinical parameters

The following clinical parameters were recorded on the same day as the transient examination: sex, age, height, BMI (calculated by dividing the weight in kg by the squared height in m), waist circumference, results of HBs antigen or anti‐HCV tests, and levels of AST, ALT, gamma‐glutamyl transpeptidase, glucose, hemoglobin A1c (HbA1c), high‐density lipoprotein (HDL) cholesterol, low‐density lipoprotein (LDL) cholesterol, and triglycerides.

The FIB‐4 index was calculated as follows:

{age × AST (U/L)}/{blood platelet count (10^9^/L) ×√ALT (U/L)}.

The APRI score was calculated as follows:

{(AST/ULN)/platelet count (× 10^9^/L)} × 100.

The NAFLD fibrosis score was calculated as follows.

−1.675 + 0.037 × age (years) + 0.094 × BMI (kg/m^2^) + 1.13× DM (yes = 1, no = 0) + 0.99 × AST (U/l)/ALT (U/;) −0.013× platelet counts (10^4^/μL) −0.66× albumin (g/dL).

The FAST score was calculated as follows:

{exp. (−1.65 + 1.07 × ln (LSM) + 2.66 × 10–8 ×CAP3–63.3 × AST – 1)}/{1 + exp. (−1.65 + 1.07 × ln (LSM) +2.66 × 10–8 × CAP3–63.3 × AST – 1)}.^30^

A FAST score of 0.35 was used as the cutoff value for advanced fibrosis.

### Dietary pattern analysis

Energy and nutrient intakes were calculated based on the results of a brief self‐administered diet history questionnaire (BDHQ), which was developed for use in large‐scale nutritional epidemiological studies. It consists of 80 questions and estimates the intake of 58 food items and over 100 nutrients.^38^

### MASLD diagnosis

Participants with fatty liver who met any of the following cardiometabolic criteria encompassing obesity/central obesity, hyperglycemia or diabetes, high blood pressure, high triglycerides, and reduced HDL cholesterol were diagnosed with MASLD.^2^ The specific criteria included a BMI ≥23 kg/m^2^ or waist circumference ≥ 94 cm for men and ≥ 80 cm for women; fasting blood glucose ≥100 mg/dL, postprandial blood glucose ≥140 mg/dL, HbA1c ≥ 5.7%, or undergoing treatment for type 2 diabetes; blood pressure ≥ 130/85 mmHg or currently undergoing antihypertensive treatment; triglycerides ≥150 mg/dL or currently undergoing treatment for dyslipidemia; and HDL cholesterol ≤40 mg/dL for men and ≤ 50 mg/dL for women. Although the waist of diagnostic criteria for MASLD is different from that of Japanese metabolic syndrome diagnostic criteria (≥94 cm for men and ≥ 80 cm for women), the compatibility rate with MASLD is reported to be more than 95%, and the diagnostic criteria for MASLD were applied in this study.^39^

### DNA preparation and SNP genotyping

SNP genotypes were determined by whole‐genome sequencing, with imputation from the Japonica Array (Toshiba, Tokyo, Japan), consisting of population‐specific SNP markers designed from the 1070 whole‐genome reference panel and TaqMan PCR.^40^, ^41^ Whole‐genome sequencing and imputation were performed by the Takara Bio Corporation (Shiga, Japan) and Toshiba Corporation, respectively. For the Japonica Array, DNA was purified from peripheral whole blood using a QIAamp®96 DNA Blook Kit (QIAGEN, Hilden, Germany) and extracted from the plasma pellets for whole‐genome sequencing. Many NAFLD susceptibility genes have been reported so far. In this study, we analyzed 13 SNPs that have been frequently reported to be associated with NAFLD in previous studies.^18^, ^42^ Participants were genotyped for 13 SNPs associated with NAFLD: PNPLA3‐rs rs738409 (risk alleles G), PNPLA3‐rs 2 896 019 (risk alleles G), LYPLAL1‐rs12137855 (risk alleles C), GCKR‐rs1260326 (risk alleles C), AGTR1‐rs3772622 (risk alleles C), PPP1R3B‐rs4240624 (risk alleles A), GATAD2A‐rs4808199 (risk alleles A), MTP‐rs1800591 (risk alleles T), Adiponetcin‐rs2241766 (risk alleles G), PEMP‐rs7496 (risk alleles T), MnSO‐rs4880 (risk alleles G), TM6SSF2‐rs58542926 (risk alleles T), and DYSF‐rs17007417 (risk alleles T).^10^, ^18^, ^19^, ^20^, ^21^, ^22^, ^23^, ^24^, ^27^, ^28^, ^29^

### Statistical analysis

Statistical analyses of clinical data were performed using the Statistical Package for the Social Sciences (SPSS) version 28.0 (SPSS Inc., Chicago, IL, USA). Continuous variables are presented as medians and interquartile ranges. Categorical variables are presented as frequencies, and continuous variables as medians, along with interquartile ranges. Chi‐square and Mann–Whitney U tests were used to compare the two groups. The risk factors for ALT levels >30 U/L in the MASLD group were analyzed using univariate and multivariate analyses. SNPs were analyzed separately for the group with two risk alleles and the group with one or no risk alleles. SNPs associated with ALT levels >30 U/L were examined using univariate and multivariate analyses adjusted for sex, age, obesity/central obesity, hyperglycemia or diabetes, high blood pressure, high triglycerides, and reduce HDL cholesterol separately for each of the 13 MASLD‐related SNPs. In addition, SNPs that were risk factors for FAST score > 0.35 were analyzed using multivariate analysis adjusted for the presence or absence of three or more cardiometabolic criteria. Statistical significance was set at *P* < 0.05.

### Ethics statement

This study was conducted in accordance with the ethical standards of the Declaration of Helsinki and approved by the Ethics Committee of Hirosaki University School of Medicine (approval number and date: 2018–012, approved on May 11, 2018). Informed consent was obtained from all of the participants.

## Results

### Participants' characteristics

Participants' characteristics are presented in Table 1. The ALT level > 30 U/L group consisted of 56 participants, accounting for 24.6% of the patients with MASLD. Compared with the ALT level < 30 U/L group, the ALT level > 30 U/L group had a lower mean age and HDL cholesterol levels and a higher mean BMI, mean waist circumference, diastolic blood pressure, and levels of triglycerides, AST, ALT, CAP, and LSM. There were no significant differences in smoking habits, exercise habits, or nutrient intake between the two groups. Among the five cardiovascular metabolic risk factors that are part of the MASLD diagnostic criteria, the ALT level > 30 U/L group had a significantly higher prevalence of obesity/central obesity, high triglycerides, and reduced HDL cholesterol. Additionally, the number of cardiovascular metabolic risk factors was significantly higher in the ALT level > 30 U/L group (three factors) than in the ALT level > 30 U/L group (two factors).

**Table 1:** The characteristics of the participants

Variables	ALT level ≤ 30 U/L group	ALT level > 30 U/L group	*P*‐value
	*n* = 172	*n* = 56	
Sex, male	50 (29.1%)	36 (64.3%)	<0.001
Age (year)	60.5 (47.3–68.0)	50.5 (37.3–63.8)	0.001
BMI (kg/m^2^)	24.1 (22.3–26.0)	26.6 (24.0–30.7)	<0.001
Waist circumference (cm)	81.5 (74.5–87.0)	88.8 (83.4–95.2)	<0.001
Fasting blood sugar (mg/dL)	94.0 (89.0–104.0)	99.5 (88.0–112.0)	0.148
HbA1c (%)	5.8 (5.6–6.1)	5.9 (5.6–6.3)	0.524
Systolic blood pressure (mmHg)	126.0 (116.0–138.8)	131.0 (121.0–143.5)	0.194
Diastolic blood pressure (mmHg)	78.0 (71.3–87.0)	82.5 (72.8–93.0)	0.064
Triglycerides (mg/dL)	88.0 (62.0–121.8)	120.5 (96.0–175.0)	<0.001
HDL cholesterol (mg/dL)	60.0 (50.0–71.0)	50.0 (46.0–61.0)	<0.001
LDL cholesterol (mg/dL)	125.0 (108.0–144.0)	121.5 (108.0–145.8)	0.936
CAP (dB/m)	267.0 (250.0–300.5)	297.0 (273.0–330.8)	<0.001
LSM (kPa)	4.3 (3.5–5.3)	5.6 (3.9–8.0)	<0.001
APRI	0.19 (0.15–0.23)	0.31 (0.25–0.43)	<0.001
FIB‐4 index	1.11 (0.73–1.46)	0.82 (0.58–1.61)	0.160
NFS	−1.46 (−2.40 to −0.54)	−2.06 (−3.30 to −0.69)	0.054
FAST score	0.034 (0.061–0.097)	0.230 (0.138–0.416)	<0.001
Aspartate aminotransferase (U/L)	20.0 (17.0–23.0)	31.5 (26.0–40.8)	<0.001
Alanine aminotransferase (U/L)	18.0 (14.0–22.0)	46.0 (37.0–65.3)	<0.001
γ‐Glutamyl TransPeptidase (U/L)	20.0 (16.0–28.0)	49.0 (31.5–85.0)	<0.001
Smoking habit	25 (14.5%)	12 (21.4%)	0.224
Exercise habit	31 (18.0%)	10 (17.9%)	0.577
Energy intake per day (kcal/day)	1726.6 (1451.4–2104.4)	1784.5 (1494.2–2152.3)	0.270
Protein intake per day (g/1000 kcal)	39.3 (35.6–44.8)	38.6 (34.2–41.3)	0.113
Fat intake per day (g/1000 kcal)	30.0 (25.7–34.2)	29.5 (25.7–33.7)	0.648
Carbohydrate intake per day (g/1000 kcal)	136.7 (123.1–148.2)	138.4 (125.7–148.4)	0.414
Cardiometabolic risk factors			
Obesity/central obesity	81 (47.1%)	38 (67.9%)	0.005
Hyperglycemia or diabetes	137 (79.7%)	42 (75.0%)	0.287
High blood pressure	103 (59.9%)	40 (71.4%)	0.081
High triglycerides	54 (31.4%)	27 (48.2%)	0.018
Reduced HDL cholesterol	28 (16.3%)	17 (30.4%)	0.020
Risk factor numbers	2	3	0.002

Data are presented as numbers (%) or median (range).

APRI, aspartate aminotransferase to platelet ratio index; BMI, body mass index; CAP, controlled attenuation parameter; FAST score, FibroScan‐AST (FAST)score lipoprotein; HDL, high‐density lipoprotein; LDL, low‐density lipoprotein; LSM, liver stiffness measure; NFS, NAFLD fibrosis score.

### Frequencies of MASLD‐associated genetic variants

The frequencies of the 13 NAFLD‐associated SNPs measured in this study are listed in Table 2. The PNPLA3 rs738409 GG genotype and rs2896019 GG genotype were approximately twice as prevalent in the ALT level > 30 U/L group compared with the ALT level ≤ 30 U/L group. On the other hand, about 16% of the ALT level ≤ 30 U/L group had the PNPLA3 rs738409 and rs2896019 GG genotypes. No significant differences were observed in the prevalence of other SNPs between the two groups. The PPP1R3B rs4240624 AA genotype was present in almost all participants, whereas the MnSO rs4880 GG, TM6SSF2 rs58542926 TT, and DYSF rs17007417 TT genotypes were rarely observed.

**Table 2:** The frequencies of the 13 MASLD‐associated SNPs measured in this study.

Variables	Genotype	ALT level ≤ 30 U/L group *n* = 172	ALT level > 30 U/L group *n* = 56	*P*‐value
PNPLA3 (rs738409)	CC	49 (28.5)	12 (21.4)	0.016
	CG	95 (55.2)	24 (42.9)	
	GG^†^	30 (17.4)	20 (35.7)	
PNPLA3 (rs2896019)	TT	49 (28.5)	12 (21.4)	0.036
	TG	95 (55.2)	26 (46.4)	
	GG^†^	28 (16.3)	18 (32.1)	
LYPLAL1 (rs12137855)	TT	0 (0.0)	0 (0.0)	0.154
	TC	19 (11.0)	11 (19.6)	
	CC^†^	153 (89.0)	45 (80.4)	
GCKR (rs1260326)	TT	61 (35.5)	16 (28.6)	0.528
	TC	69 (40.1)	27 (48.2)	
	CC^†^	42 (24.4)	13 (23.2)	
AGTR1 (rs3772622)	TT	66 (38.4)	18 (32.1)	0.423
	TC	78 (45.3)	31 (55.4)	
	CC^†^	28 (16.3)	7 (12.5)	
PPP1R3B (rs4240624)	GG	0 (0.0)	0 (0.0)	1.000
	GA	1 (0.6)	0 (0.0)	
	AA^†^	171 (99.4)	56 (100)	
GATAD2A (rs4808199)	GG	95 (55.2)	30 (53.6)	0.931
	GA	67 (39.0)	22 (39.3)	
	AA^†^	10 (5.8)	4 (7.1)	
MTP (rs1800591)	GG	112 (65.1)	42 (75.0)	0.3779
	GT	53 (30.8)	12 (21.4)	
	TT^†^	7 (4.1)	2 (3.6)	
Adiponectin (rs2241766)	TT	84 (48.8)	29 (51.8)	0.508
	TG	74 (43.0)	25 (44.6)	
	GG^†^	14 (8.1)	2 (3.6)	
PEMT (rs7496)	CC	145 (84.3)	47 (83.9)	0.698
	CT	26 (15.1)	8 (14.3)	
	TT^†^	1 (0.6)	1 (1.8)	
MnSO (rs4880)	AA	135 (78.5)	38 (67.9)	0.149
	AG	35 (20.3)	18 (32.1)	
	GG^†^	2 (1.2)	0 (0.0)	
TM6SSF2 (rs58542926)	CC	150 (87.2)	47 (83.9)	0.507
	CT	20 (11.6)	9 (16.1)	
	TT^†^	2 (1.2)	0 (0.0)	
DYSF (rs17007417)	CC	125 (72.7)	36 (64.3)	0.040
	CT	38 (22.1)	20 (35.7)	
	TT^†^	9 (5.2)	0 (0.0)	

^†^ Risk alleles of genotype.

Data are presented as numbers (%).

### Risk factors for ALT levels >30 U/L in MASLD

In the MASLD group, the risk factors for ALT levels >30 U/L, as determined by univariate analysis, were male sex, younger age, and the following cardiovascular metabolic risk factors: obesity/central obesity, high triglycerides, and reduced HDL cholesterol. In the multivariate analysis, male sex, younger age, and high triglyceride levels were significant risk factors for ALT levels >30 U/L (Table 3).

**Table 3:** Univariable and multivariate analyses of risk factors for ALT>30 in MASLD

	Univariable				Multivariable			
	OR	95% CI		*P*‐value	OR	95% CI		*P*‐value
Male	4.39	2.32	8.31	<0.001	4.15	2.06	8.36	<0.001
Age	0.96	0.94	0.98	<0.001	0.97	0.94	0.99	0.008
Obesity/central obesity	2.37	1.26	4.48	0.008	1.92	0.95	3.90	0.071
Hyperglycemia or diabetes	0.77	0.38	1.56	0.463				
High blood pressure	1.67	0.87	3.22	0.123				
High triglycerides	2.03	1.10	3.76	0.024	2.41	1.18	4.91	0.015
Reduced HDL cholesterol	2.24	1.11	4.51	0.024	1.79	0.78	4.13	0.172
Smoking habit	1.60	0.75	3.45	0.227				
Exercise habit	0.99	0.45	2.17	0.978				
Energy intake per day	1.00	1.00	1.00	0.162				
Carbohydrate intake per day	1.01	0.99	1.02	0.440				
Protein intake per day	0.96	0.92	1.01	0.090				
Fat intake per day	0.99	0.94	1.04	0.674				

CI, confidence interval; OR, odds ratio.

The NAFLD‐associated SNPs identified as risk factors for ALT levels >30 U/L are shown in Table 4. In both univariate (Model 1) and multivariate analyses adjusted for sex, age, and high triglyceride levels (Model 2), the GG genotypes of PNPLA3 rs2896019 and rs738409 were significant risk factors for ALT levels >30 U/L.

**Table 4:** Univariate and multivariate analyses of risk factors for ALT>30 in MASLD

Variables	Risk alleles of genotype	Model 1				Model 2			
		OR	95% CI		*P*‐value	OR	95% CI		*P*‐value
PNPLA3 (rs738409)	GG	2.63	1.34	5.16	0.005	3.19	1.44	7.07	0.004
PNPLA3 (rs2896019)	GG	2.44	1.22	4.86	0.012	3.11	1.37	7.06	0.007
LYPLAL1 (rs12137855)	CC	0.51	0.23	1.15	0.103	0.53	0.20	1.41	0.204
GCKR (rs1260326)	CC	0.94	0.46	1.91	0.855	0.94	0.42	2.13	0.887
AGTR1 (rs3772622)	CC	0.74	0.30	1.79	0.497	0.58	0.21	1.63	0.303
PPP1R3B (rs4240624)	AA	–	–	–	–	–	–	–	–
GATAD2A (rs4808199)	AA	1.25	0.38	4.14	0.719	0.74	0.17	3.19	0.684
MTP (rs1800591)	TT	0.87	0.18	4.33	0.868	1.75	0.27	11.3	0.558
Adiponectin (rs2241766)	GG	0.42	0.09	1.90	0.259	0.35	0.07	1.82	0.211
PEMT (rs7496)	TT	3.11	0.19	50.50	0.425	2.51	0.08	74.2	0.595
MnSO (rs4880)	GG	–	–	–	–	–	–	–	–
TM6SSF2 (rs58542926)	TT	–	–	–	–	–	–	–	–
DYSF (rs17007417)	TT	–	–	–	–	–	–	–	–

Model 1: Unadjusted; Model 2: Adjusted for sex, age, obesity/central obesity, hyperglycemia or diabetes, high blood pressure, high triglycerides, and reduced HDL cholesterol.

CI, confidence interval; OR, odds ratio.

### Association between PNPLA3 SNPs and liver fibrosis

The association between the rs738409 and rs2896019 SNPs of PNPLA3 and liver fibrosis‐related markers are shown in Table 5. The GG genotypes of PNPLA3 rs738409 and rs2896019 had higher FAST and APRI scores than the CC + CG and TT + TG genotypes. The PNPLA3 rs2896019 GG genotype also had a higher LSM level than the TT + TG genotype.

**Table 5:** The association between the PNPLA3 rs738409 or rs2896019 and liver fibrosis‐related markers

PNPLA3 rs738409			
Variables	CC/CG	GG	*P*‐value
	*n* = 178		*n* = 50
FAST score	0.07 (0.04–0.13)	0.14 (0.04–0.22)	0.011
APRI	0.20 (0.16–0.26)	0.25 (0.18–0.32)	0.004
FIB4 index	1.02 (0.63–1.47)	1.03 (0.79–1.48)	0.334
NFS	−1.56 (−2.67 to −0.54)	−1.68 (−2.27 to −0.77)	0.830
LSM	4.4 (3.5–5.4)	4.8 (3.7–6.1)	0.168

PNPLA3 rs2896019			
Variables	TT/TG	GG	*P*‐value
	*n* = 182	*n* = 46	
FAST score	0.07 (0.04–0.13)	0.13 (0.04–0.22)	0.013
APRI	0.20 (0.16–0.26)	0.24 (0.18–0.31)	0.009
FIB4 index	1.02 (0.63–1.48)	1.02 (0.79–1.41)	0.632
NFS	−1.57 (−2.67 to −0.55)	−1.60 (−2.26 to −0.60)	0.882
LSM	4.4 (3.5–5.4)	4.9 (3.8–6.2)	0.049

Data are presented as median (range).

APRI, aspartate aminotransferase to platelet ratio index; FAST score, FibroScan‐AST(FAST)score; LSM, liver stiffness measure; NFS, NAFLD fibrosis score.

Multivariate analysis to identify risk factors for FAST score > 0.35 revealed that the GG genotypes rs738409 and rs2896019 were significant risk factors, independent of having three or more cardiovascular metabolic risk factors (Table 6).

**Table 6:** Risk factors for Fast score > 0.35 in PNPLA3 rs738409 and rs2896019 after adjusting for having three or more cardiovascular metabolic risk factors

Variables		OR	95% CI		*P*‐value
PNPLA3 (rs738409)	GG type	2.87	1.06	7.78	0.038
PNPLA3 (rs2896019)	GG type	3.28	1.20	8.95	0.021

CI, confidence interval; OR, odds ratio.

## Discussion

This study revealed that male sex, younger age, and high triglyceride levels were associated with ALT levels >30 U/L in patients with MASLD. Furthermore, the GG genotype of the PNPLA3 SNPs rs2896019 and rs738409 was found to be associated with ALT levels >30 U/L and was a risk factor for advanced liver fibrosis independent of cardiovascular metabolic risk factors in MASLD.

In Japan, an ALT level > 30 U/L was declared as the cutoff value for general medical examinations in 2023 to promote the early detection and treatment of liver disease. Normal ALT levels are generally considered to be 30 U/L worldwide.^43^, ^44^ Our multivariate analysis revealed that male sex, younger age, and high triglyceride levels, a cardiovascular metabolic risk factor, were significantly associated with ALT levels >30 U/L. However, previous studies have suggested that the ALT cutoff value should be lower in women than in men. For instance, the American Association for the Study of Liver Diseases recommends a cutoff value of 19 U/L for women.^44^ Moreover, hepatic function declines with age, leading to decreased albumin levels and increased bilirubin levels. Consistent with these changes, ALT levels reportedly decrease with age.^45^, ^46^ In this study, 16% of all project participants were MASLD patients with ALT ≤30 U/L, which could be influenced by gender and age. In contrast, our study did not identify cardiovascular metabolic risk factors other than high triglyceride levels, alcohol consumption, smoking habits, or nutrient intake as factors associated with ALT levels >30 U/L. Although these factors significantly contribute to hepatic steatosis, our results suggest that their impact on liver inflammation is not substantial.

Among the 13 MASLD‐related SNPs assessed in this study, only PNPLA3 (rs738409 and rs2896019) was significantly associated with ALT levels >30 U/L in the MASLD group. The prevalence of the GG genotypes of PNPLA3 rs738409 and PNPLA3 rs2896019 was 21.9% and 20.2% in the MASLD group, respectively. Previous studies have shown that the prevalence of the PNPLA3 rs738409 GG and rs2896019 genotypes was 38.3% and 41.3%, respectively, in Japanese patients with NAFLD, which were higher than those in our study participants.^11^, ^15^ This difference may be attributed to several factors, including different diagnostic criteria for patients with MASLD. Unlike previous studies that diagnosed fatty liver using liver biopsy, we used the FibroScan. Moreover, while a CAP value of 232.5 dB/m has been used as a cutoff for the diagnosis of SLD, some studies suggest a more stringent cutoff of 248 dB/m for SLD.^37^, ^47^ These may have been the reasons for the different results. PNPLA3 is highly expressed in hepatocyte lipid droplets and possesses lipase activity. Genetic abnormalities in PNPLA3 have been linked to the accumulation of neutral fat in hepatocytes.^48^ Additionally, numerous studies have implicated PNPLA3 SNPs in NAFLD development and progression.^9^, ^10^, ^18^ In particular, the exonic SNP rs738409 (C > G) in PNPLA3 is associated with NAFLD in several ethnicities, including Japanese.^10^, ^11^ In contrast, rs2896019 (T > G), an intronic SNP in PNPLA3, has fewer published studies but has been associated with fatty liver diseases in Asians.^10^, ^15^ Furthermore, the rs738409 PNPLA3 GG genotype was associated with increased AST and ALT levels.^49^, ^50^ Individuals with the G variant of rs2896019 have higher ALT levels than those with the TT genotype.^17^, ^49^ Our results support these previous findings and suggest that patients with MASLD having ALT levels >30 U/L are more likely to carry PNPLA3 risk alleles. On the other hand, 16% of the ALT level ≤ 30 U/L group had the PNPLA3 rs738409 and rs2896019 GG genotypes. One possible reason for this was that the majority of GG genotype MASLD with ALT ≤30 U/L were female, 76.7% for PNPLA3 rs738409 and 82.1% for rs2896019. The median ALT level of 18.5 U/L in women in this study was significantly lower than that of men (27.5 U/L), suggesting that ALT levels are lower in women than in men, even with the PNPLA3 GG genotype.

While a FAST score of ≥ 0.35 is considered the cutoff value for advanced fibrosis in NASH, multivariate analysis adjusted for cardiovascular metabolic risk factors revealed that the GG genotype of PNPLA3 rs738409 and rs2896019 remained a significant risk factor for FAST score > 0.35 in this study. Meta‐analyses have reported that G allele carriers of PNPLA3, particularly rs738409, are strongly associated with fibrosis in patients with NASH/NAFLD.^51^, ^52^ Moreover, PNPLA3 rs738409 facilitates liver fibrosis progression due to the loss of retinyl palmitate lipase activity and impaired retinol production.^53^ Although the mechanism by which PNPLA3 rs2896019 promotes liver fibrosis remains to be elucidated, a mechanism similar to that of rs738409 is possible. In this study, we observed an association between PNPLA3 and both the FAST and APRI scores, whereas no association was found with other fibrosis markers, such as the FIB‐4 index and NFS. Additionally, PNPLA3 G allele carriers have elevated levels of the liver enzymes AST and ALT.^17^, ^49^, ^50^ Both the FIB‐4 index and NFS use ALT as the denominator in their formulas. In this study, the PNPLA3 GG genotype was associated with significantly higher ALT levels than the CC/CG and TT/TG genotypes (data not shown). Therefore, it is possible that the PNPLA3 GG genotype resulted in a lower FIB‐4 index and NFS values, potentially obscuring any significant differences. Additionally, the GG genotypes of PNPLA3 rs738409 and rs2896019 were suggested to be associated with a higher risk of fibrosis progression than other NAFLD‐related SNPs. However, we did not identify any SNP other than PNPLA3 rs738409 and rs2896019 as risk factors for ALT levels >30 U/L. All SNPs assessed in this study were previously reported to be associated with NAFLD/NASH.^9^, ^10^, ^11^, ^12^, ^13^, ^14^, ^15^, ^16^, ^17^, ^18^, ^19^, ^20^, ^21^, ^22^, ^23^, ^24^, ^25^, ^26^, ^27^, ^28^, ^29^ Moreover, SNPs other than PNPLA3 were associated with hepatic lipidification but had no or very mild effects on increasing ALT levels and liver fibrosis, suggesting that they may not have been associated in this study.

Nevertheless, this study had some limitations. First, fatty liver and liver fibrosis were diagnosed using FibroScan instead of a liver biopsy because an invasive liver biopsy, which is conducted as part of a general population health check, was not feasible in this study. Second, because SNPs vary by ethnicity, it is not appropriate to generalize the results of this study, which were conducted in one region of Japan, to other ethnicities. Furthermore, it has been reported that Asians, including Japanese, have a higher prevalence of lean MASLD.^54^, ^55^ In our study, lean MASLD accounted for approximately half (47.8%) of all MASLD patients. The frequency of the PNPLA3 rs738409 and rs2896019 GG genotypes in lean MASLD patients was 22.0% and 19.3%, respectively, which was generally similar to the overall GG genotype frequency in MASLD patients. Third, MASLD is a polygenic disease, but we did not calculate a polygenic score. Calculating the polygenic score in future studies may help to elucidate the pathogenesis of MASLD.

In conclusion, individualized treatment for patients with MASLD is important due to the influence of various factors on MASLD progression, including genetic factors and lifestyle diseases.

## Ethics statement

This study was conducted in accordance with the ethical standards of the Declaration of Helsinki and approved by the Ethics Committee of Hirosaki University School of Medicine (approval number and date: 2018–012, approved on May 11, 2018).

## Patient consent statement

Informed consent was obtained from all participants.

## Data Availability

Raw data were generated at Hirosaki University Graduate School of Medicine. Derived data supporting the findings of this study are available from the corresponding author on request.
